# Sequence Analysis of the Capsid Gene during a Genotype II.4 Dominated Norovirus Season in One University Hospital: Identification of Possible Transmission Routes

**DOI:** 10.1371/journal.pone.0115331

**Published:** 2015-01-15

**Authors:** Barbara Juliane Holzknecht, Kristina Træholt Franck, Rikke Thoft Nielsen, Blenda Böttiger, Thea Kølsen Fischer, Jannik Fonager

**Affiliations:** 1 Department of Microbiological Diagnostics and Virology, Statens Serum Institut, Copenhagen, Denmark; 2 Department of Clinical Microbiology, Copenhagen University Hospital Herlev, Herlev, Denmark; 3 Research Unit for Clinical Microbiology, University of Southern Denmark, Odense, Denmark; 4 Department of Clinical Microbiology, Odense University Hospital, Odense, Denmark; 5 Medical Microbiology, Department of Laboratory Medicine Malmö, Lund University, Malmö, Sweden; University of California, San Francisco, UNITED STATES

## Abstract

Norovirus (NoV) is a leading cause of gastroenteritis and genotype II.4 (GII.4) is responsible for the majority of nosocomial NoV infections. Our objective was to examine whether sequencing of the capsid gene might be a useful tool for the hospital outbreak investigation to define possible transmission routes. All NoV positive samples submitted from one university hospital during the 2007/8 season were selected. Genotyping of selected samples by partial polymerase gene sequencing had shown that the majority belonged to the GII.4 variant Den Haag 2006b and had identical polymerase sequences. Sequences of the capsid gene (1412 nucleotides) were obtained from the first available sample from 55 patients. From six immunocompromised patients with persistent infections a second sample was also included. As a control for a point-source outbreak, five samples from a foodborne outbreak caused by the same GII.4 variant were analyzed. Forty-seven of the inpatients (85%) were infected with the GII.4 variant Den Haag 2006b. Phylogenetic analysis of the Den Haag 2006b sequences identified four distinct outbreaks in different departments and a fifth outbreak with possible inter-department spread. In addition, a more heterogeneous cluster with evidence of repeated introductions from the community, but also possible inter-department spread was observed. In all six patients with paired sequences, evidence for *in vivo* evolution of the virus was found. Capsid gene sequencing showed substantial sequence variation among NoV GII.4 variant Den Haag 2006b strains from one single institution during a nine months’ period. This method proved useful to understand the local epidemiology and, when used promptly, has the potential to make infection control measures more targeted.

## Introduction

Norovirus (NoV) is a leading cause of acute gastroenteritis worldwide, causing both outbreaks and sporadic cases [1–6]. It is a positive-sense single-stranded non-enveloped RNA-virus belonging to the family *Caliciviridae*. As other RNA-viruses, it is characterized by a high mutation rate, leading to the rapid emergence of genetically diverse strains. At least five genogroups can be distinguished and subdivided into approximately 30 genotypes [1, 7]. The genotype II.4 (GII.4) is responsible for the majority of infections in healthcare settings [3]. Novel GII.4 variants emerge periodically and spread in a pandemic manner [8, 9] and there is growing evidence that this is due to antigenic drift in response to selective pressure from the host population [10–13].

In areas with a temperate climate, NoV infections occur mainly during the winter season. Outbreaks occur frequently in semi-closed or closed environments such as hospitals, schools and cruise ships [3] and can cause major health challenges in health care settings, in particular if effective control measures are not rapidly implemented.

Although symptomatic NoV infection in immunocompetent patients is usually limited to a few days, NoV can cause persistent infections in immunosuppressed patients accompanied by substantial *in vivo* evolution of the virus, leading to the co-existence of quasispecies [14–16].

For the detection and partial sequencing of NoV RNA, primers targeting the polymerase gene [17–19], the capsid gene [20] and the junction of the open reading frame (ORF) 1 and ORF2 [21, 22] have been developed. These methods have been useful in outbreak investigation, especially in determining whether an accumulation of NoV cases is composed of several different genotypes, likely reflecting individual introductions and sometimes called a pseudo outbreak [23]. However, sequencing of longer parts of the capsid gene, especially the hypervariable P2 domain, provides more detailed information and has recently been used to study transmission routes [24–28]. Whole genome sequencing using next generation sequencing technology has also proved feasible and useful in outbreak investigation [29].

Our objective was to investigate the possible transmission routes of GII.4 infections in one hospital during a nine months’ period by sequencing of the capsid gene. The aim was to assess if this method can be used to differentiate sustained nosocomial transmission from repeated introductions of different strains from the community and to distinguish between distinct outbreaks in different hospital departments and inter-department spread.

## Materials and Methods

### Setting and sampling

All stool samples positive for NoV and submitted from of a 770-bed university hospital in the Copenhagen area, Denmark, to the virological department at Statens Serum Institut for diagnosis during November 2007 to July 2008, were identified from the laboratory database. One hundred-and-fifty-three samples from 105 patients had been submitted from seven different departments. Capsid and/or polymerase typing demonstrated that the majority (95% of 60 samples examined) belonged to GII.4.

The first available sample from each patient was included for analysis with a GII.4 specific reverse transcription polymerase chain reaction (RT-PCR). For 29 patients, samples were no longer available and for another 20 patients the GII.4 specific capsid RT-PCR was negative. One sample was excluded since previous genotyping had shown a genotype GI.3. Thus, sequences from a total of 55 patients were analyzed. From six of these patients, additional sequences were obtained from follow-up samples taken at least 14 days after the first sample. In addition, five samples from a foodborne outbreak caused by GII.4 variant Den Haag 2006b, occurring in 2008, were included in the study in order to analyze the capsid gene sequence variation in a known point-source outbreak. In total, 66 capsid sequences were obtained and analyzed.

For 22 of the inpatients with available capsid sequences and for the five samples from the foodborne outbreak, polymerase gene sequences were available from previous genotyping.

### Clinical data

Electronic patient records were reviewed for admission data, onset of gastrointestinal symptoms and presence of immunosuppressive disorders or prescription of immunosuppressive therapy.

### Laboratory methods

NoV diagnostics and capsid and/or polymerase typing was performed as previously described [30].

For the GII.4 specific RT-PCR; RNA was extracted from 10% stool suspensions, which had been kept frozen at-80°C, with the MagNAPure LC (Roche Diagnostics, Hvidovre, Denmark). A NoV GII.4-specific nested RT-PCR amplifying 1571 nucleotides (nt) of the capsid gene (ORF2) was performed using primers as previously described [31]. Reverse transcription and the first PCR reaction was carried out in a volume of 25 µl (concentration of dNTP 0.4 mM, primers 0.6 µM, and RNAse inhibitor 10 U) using the QIAGEN OneStep RT-PCR Kit (Qiagen Nordic Denmark) and the following cycling conditions: 42°C for 1 h, 94°C for 15 min, 40 cycles of 94°C for 30 s, 50°C for 30 s, 72°C for 2 min each and final extension at 72°C for 10 min. The second PCR reaction was carried out in a total volume of 25 µl (concentration of MgCL_2_ 4 mM, dNTP 0.4 mM, primers 0.6 µM), using GeneAmp 10X PCR Buffer II, MgCl_2_ and AmpliTaq DNA Polymerase (Applied Biosystems, Naerum, Denmark) and cycling conditions: 95°C for 5 min, 35 cycles of 95°C for 30 s, 52°C for 30 s, 72°C for 2 min and final extension at 72°C for 5 min. The PCR product was sequenced with the PCR primers, eventually supplemented with sequencing primers [31] using Big Dye Terminator kit 3.1 (Applied Biosystems, Naerum, Denmark).

### Sequence analysis

Sequences were quality assessed and assembled using the BioNumerics version 6.6 software (Applied Maths, Sint-Martens-Latem, Belgium). BioNumerics has an in-built algorithm for sequence quality assessment and automatically discards low quality sequences before assembly. In addition, all sequence chromatograms were inspected manually and finally, all sequences were compared with publically available sequences deposited in GenBank via BLASTN and through manual alignments. For polymerase gene sequences, the full available sequences with a length ranging from 226 to 285 nucleotides (nt) were analyzed. For capsid gene sequences, a 1412 nucleotide (nt) region, corresponding to nt position 110 to 1521 of the reference sequence, was shared by all sequences and was used for further analysis. The norovirus sequence Hu/GII.4/Shellharbour/NSW696T/2006/AUS (GenBank no. EF684915.2) [32] was used as a reference. The P2 region was defined as described by Vega et al. [33], ranging from nt position 820 to 1254 of the reference capsid sequence. For genotype assignment, the online norovirus typing tool was used (http://www.rivm.nl/mpf/norovirus/typingtool) [34].

For further analysis, sequence alignment, conceptual translation to amino acid sequences, estimates of evolutionary divergence and phylogenetic analysis were performed in *MEGA* version 6 [35]. Sequence variation was defined as nucleotide changes among the sequences in this study or the detection of mixed bases (i.e. more than one peak at the same position in the sequence chromatogram in sequences with otherwise good quality). Sites with mixed bases were not included in the estimates of evolutionary divergence and phylogenetic analysis. For the phylogenetic analysis, the Neighbor Joining (NJ) method (Jukes-Cantor model, bootstrap test with 1,000 replicates, uniform rates among sites, complete deletion of missing data) was used. To evaluate robustness of the phylogenetic analysis, it was repeated by constructing a Maximum Likelihood tree (General Time Reversible model, bootstrap test with 1,000 replicates, uniform rates among sites, complete deletion of missing data, otherwise default settings). Key amino acid positions in the P2 region which have been shown to be involved in receptor binding or to be predicted epitopes [36] were manually identified by alignment with the conceptually translated reference sequence.

### Nucleotide sequence accession numbers

All sequences were submitted to GenBank. Capsid sequences have accession numbers KJ144938 to KJ145003, and partial polymerase sequences have accession numbers KJ956701 to KJ956727. A detailed assignment is available as supplemental material (S1 Table).

### Statistical analysis and Graphics

The computer program “R: A language and environment for statistical computing”, version 3.0.2 (R Foundation for Statistical Computing, Vienna, Austria; http://www.R-project.org/) was used for statistical analysis. For comparison of categorical data, Fisher’s exact test was used if expected values were <5, otherwise the Chi-squared test was used. P-values ≤0.05 were considered statistically significant.

GraphPad Prism Version 5.04 (GraphPad Software, La Jolla, CA, USA) was used for graphics.

### Ethics statement

The study was approved by the Danish Data Protection Agency (Record no. 2012–54–0202). Because of the retrospective design of the study, no written or verbal informed consent could be given by the patients. That being the case, access to patient records has to be approved by the Danish Health and Medicines Authority according to Danish law. This approval was obtained (Record no. 3-3013-292/1/). After retrieving information from electronic patient records data was anonymized by the research team for further analysis.

According to the “Danish Act on Research Ethics Review of Health Research Projects” this study does not require approval by the ethics committees, as it is considered a quality development project. This was confirmed by the Committees on Health Research Ethics for the Capital Region of Denmark in a specific waiver of approval (H-6-2014-FSP-055).

## Results

### Distribution of GII.4 variants

Capsid sequencing showed that the majority of the inpatients (47 of 55) were infected with the Den Haag 2006b variant and three and five inpatients were infected with the Osaka 2007 variant and the Apeldoorn 2007 variant, respectively. The foodborne outbreak was caused by the Den Haag 2006b variant.

In all 24 samples, where a genotype could be assigned from the polymerase sequence, it was congruent with the capsid genotype. Thus, no recombinants were detected.

### Capsid sequence variation between and within variants

Comparing all 66 capsid sequences, nucleotide changes were observed in 238 of 1412 sites (17%). There were no deletions or insertions. The estimated evolutionary divergence between the three GII.4 variants is available as supporting information (S1 Fig.).

Between four of the five sequences belonging to the Apeldoorn 2007 variant, no nucleotide changes were found. These samples were from patients in the same department and also temporarily related, while the fifth sequence differed by one nucleotide and was epidemiologically unrelated (sample obtained three months later in another department). Two sequences belonging to the Osaka 2007 variant did not show any nucleotide changes and had been obtained from patients in the same department taken with one day’s interval. The third differed by two nucleotides and was obtained two weeks earlier in another department, suggesting that this case was not related to the other two.

Among the 58 Den Haag 2006b sequences, nucleotide changes were detected in 79 sites (6%), 31 of which were located in the P2 region. The number of nucleotide substitutions was higher within the P2 region (31 of 435 nt, 7%) than outside (48 of 977 nt, 5%), but this difference was not statistically significant (Chi-squared test, p = 0.12). However, substitutions in the codons of the P2 region were significantly more often located in the first or second position of the codon (45% of nucleotide substitutions in P2, 15% outside P2, Chi-squared test, p = 0.006) and transversions (as opposed to transitions) were also significantly more common (32% of nucleotide changes in P2, 4% outside P2, Fischer’s Exact test, p = 0.001). As both of these observations are associated with non-synonymous nucleotide substitutions, 14 of the 18 amino acid changes occurred in the P2 region (Chi-squared test, p<0.001). The 18 non-synonymous substitutions resulted in 17 amino acid alterations, 5 of which were located in key amino acid positions in the P2 region [36] (marked with asterisks in Table 1).

**Table 1:** Amino acid substitutions in the 58 translated GII.4 Den Haag 2006b sequences.

**Reference position ^a^**	**Amino acid substitution ^b^**
144	Ile / Met
244	Ile / Thr
245	Pro / Ser
297*	Arg / His
305	Ser / Ala
317	Ile / Thr
323	Thr / Ala
338	Thr / Ser
340*	Gly / Ala
355*	Ser / Gly
357	Pro / Asp
372*	Glu / Asp
376	Glu / Val
378	His / Leu
398	Asn / Ser
407*	Ser / Asn
501	His / Tyr

^a^ In relation to reference capsid sequence (GenBank Accession Nr. EF684915.2). Key amino acid positions [36] are marked with asterisks.

^b^ More common / less common.

To exclude the possibility of RT-PCR introduced nucleotide changes or sequencing mistakes, cDNA synthesis, PCR and sequencing was repeated for 5 samples with unique substitutions not shared with any of the other sequences. This repeated testing showed 100% reproducibility for a total of ten nucleotide substitutions.

In addition to the above described nucleotide changes, a total of 47 mixed bases were detected in 15 sequences at 39 different positions. These mixed bases indicate quasispecies, as a mixed signal would be expected, if more than one viral strain has been co-PCR amplified and co-sequenced. The 15 sequences were from samples of 12 different patients, ten of which were receiving immunosuppressive therapy (one cardiology patient receiving high dose prednisolone, one nephrology patient receiving immunosuppressive therapy after renal transplantation and eight hematology patients receiving chemotherapy).

### Phylogenetic analysis and molecular epidemiology

The first capsid sequence from each of the 55 inpatients, the five samples from the foodborne outbreak and the reference sequence were included in the phylogenetic analysis.

**Figure 1:**
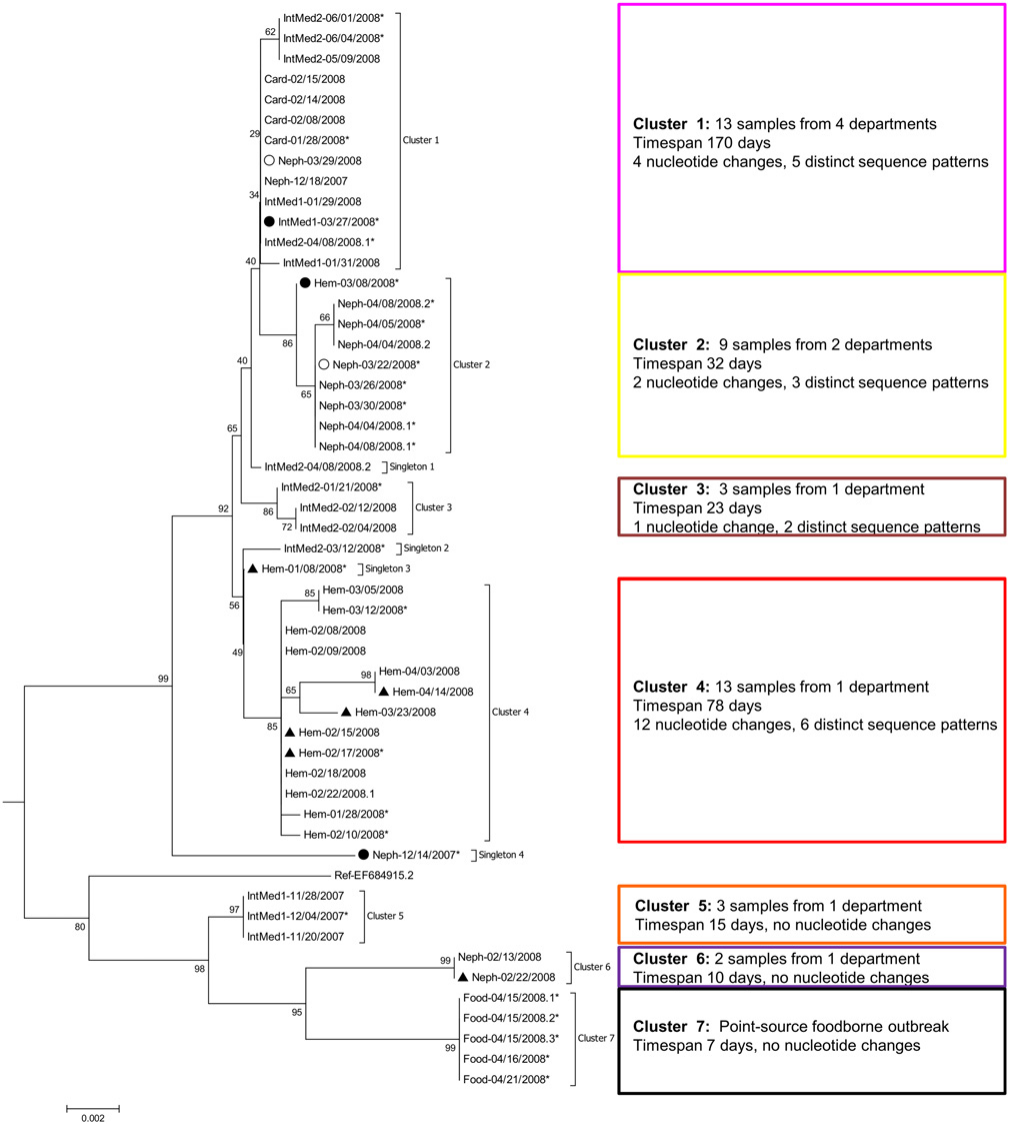
Phylogenetic tree of 53 NoV genotype II.4 variant Den Haag 2006b capsid sequences. All capsid sequences (first sample per patient) were aligned and a Neighbor Joining tree was constructed and rooted on the branch between the Den Haag 2006b variant and the Osaka 2007 and Apeldoorn 2007 sequences. The subtree of the 2006b variant is displayed here. Sites with mixed bases were ignored for phylogenetic analysis. Sequence names represent hospital department (Hem: hematology, Neph: nephrology, IntMed1/2: internal medicine 1/2, Card: cardiology, Food: foodborne outbreak) and date of sampling (mm/dd/yyyy), eventually followed by a serial number to ensure uniqueness. Sequences from patients with symptom onset within 48 hours after hospital admission are marked with a circle (filled circle: no previous contact to hospital, empty circle: frequent contact to hospital due to hemodialysis). Sequences from the first samples from six patients with persistent diarrhea are marked with a filled triangle. Sequences from samples with an available polymerase gene sequence from previous genotyping (n = 27) are marked with asterisks and phylogenetic analysis of this subgroup is shown in Fig. 4. On the right hand side of the tree epidemiological information is given as well as numbers of nucleotide changes within the cluster and the number of distinct sequence patterns included in the cluster.

Fig. 1 shows the Neighbor Joining subtree of the Den Haag 2006b capsid sequences. There were 23 distinct sequence patterns which formed seven clusters and four singletons. Clusters 2 to 7 were supported by bootstrap values >80. Cluster 1 was only supported by a bootstrap value of 36, but as it was comprised of five distinct sequence patterns only differing by four nucleotide changes it was considered a cluster in this study. When the phylogenetic analysis was repeated with the Maximum Likelihood method, the separation in these seven clusters and four singletons did not change (S2 Fig.). The estimated evolutionary divergence between the different clusters and singletons is available as supporting information (S1 Fig.).

**Figure 2:**
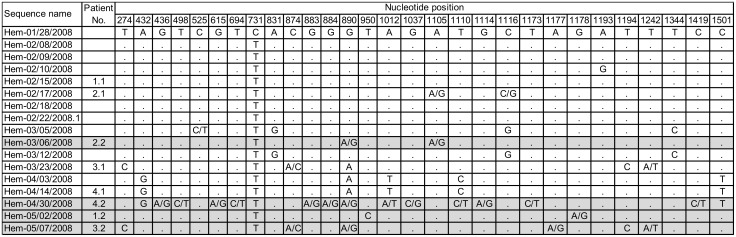
Sequence variation in cluster 4 of the GII.4 Den Haag 2006b variant. Seventeen sequences from cluster 4 are shown, including follow up samples from four patients with chronic NoV infection. Sequences are sorted chronologically and nucleotide positions with mixed bases and/or nucleotide changes are shown. Sequence names indicate department (Hem: hematology), sample date (mm/dd/yyyy) and eventual serial number. For patients with chronic NoV infections, a patient number is given followed by “.1” for first sample and “.2” for second sample (marked with grey background). The nucleotide position is given in relation to the reference capsid sequence from GenBank Accession Nr. EF684915.2. Period indicates the same nucleotide as in the first sample (Hem-01/28/2008).

Cluster 7 represented the foodborne point source outbreak and these five sequences were identical. Four of the six hospital clusters were locally confined to one department. The three smallest clusters (3, 5 and 6) were restricted to one department each and to a period of less than one month and the sequences differed by no more than one nucleotide substitution. Thus, in these clusters the molecular epidemiology confirmed the epidemiological relation and added the information, that each of these clusters was due to a new introduction of the virus into the hospital environment. Cluster 4 included 13 samples from the hematology department over a period of 78 days. Six distinct sequence patterns differing at 12 sites (0.8%) were observed. A more detailed description of the sequence variation in this cluster, taking into account mixed bases and follow up samples from four of the patients, is given in Fig. 2 and described more detailed in the next section.

The clusters 1 and 2 included sequences from samples from different departments. Cluster 2 was composed of sequences from eight samples obtained from the nephrology department and one from the hematology department. The earliest of the nephology samples was taken one day after admission in a patient undergoing chronic hemodialysis. The ninth sample was from the hematology department and was taken at the patient’s admission two weeks earlier. Thus, this cluster could have been caused by either two separate introductions or inter-department spread of the virus. Cluster 1 was epidemiologically more heterogeneous, with sequences of samples obtained from four different departments. The first sample (Neph-12/18/2007) was taken in the nephrology department in December 2007. About two months later, four samples belonging to the same cluster were taken in the cardiology department within three weeks; one of these samples differed by one nucleotide from the other three. Another two samples in this cluster were taken in the internal medicine department 1 within three days after the first sample from the cardiology department (IntMed1-01/29/2008, IntMed1-01/31/2008). One of these had an identical sequence compared to the three cardiology sequences and the other one differed by one nucleotide. This indicates that transmission between these two departments has occurred. Six weeks after the last sample was taken in the cardiology department, a sample taken from a patient in the internal medicine department 1 (IntMed1-03/27/2008) revealed a sequence differing by one nucleotide. As this sample was taken within 48 hours after admission in a patient without previous hospital contact, this most likely represents a new introduction of the virus into the hospital environment. A sequence obtained from a sample taken two days later from a chronic hemodialysis patient within 48 hours after admission (Neph-03/29/2008) and a third sequence taken two weeks later from a patient in the internal medicine department 2 (IntMed2-04/08/2008.1) were identical. Inter-department spread could have occurred here. Again one month later, three samples from the internal medicine department 2 yielded identical sequences differing from the last mentioned ones by one nucleotide substitution. This could either represent a new introduction or an ongoing outbreak in that department. Taken together, in cluster one, most likely several new introductions have taken place, but inter-department spread is also likely to have occurred (at least between the cardiology department and the internal medicine department 1).

One sample with a sporadic sequence (“singleton 1”) was taken on the same day and in the same department as a sample from cluster 1 (IntMed2-04/08/2008.1). The sequences from these two samples differed only by one nucleotide substitution (represented by the short horizontal branch length), making nosocomial transmission very likely and indicating that a more reasonable classification of this sequence would be in cluster 1.

**Figure 3:**
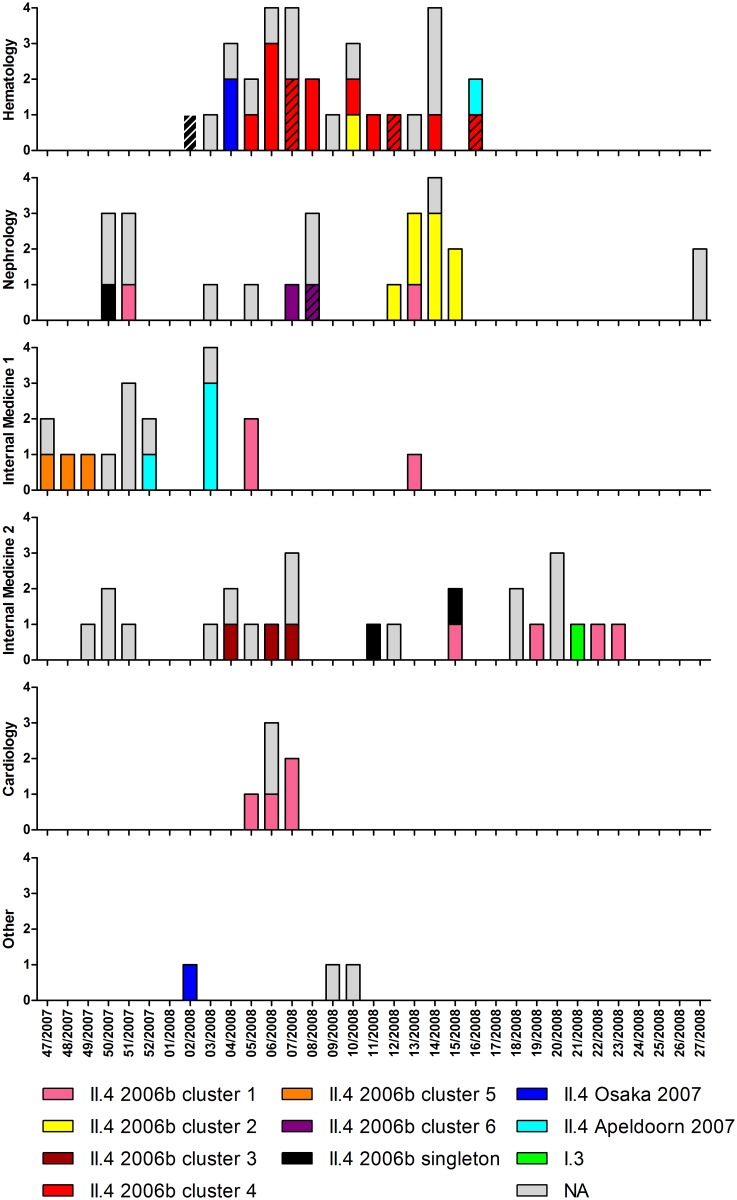
Local and temporal distribution of genotypes, variants and GII.4 2006b clusters. The first sample per inpatient is shown (n = 105). Units on vertical axes represent numbers of patients. NA (n = 49): no sequence information available (n = 29: sample not stored, n = 20: GII.4 specific PCR negative). The sequences from the first samples from six patients with persistent diarrhea are marked with diagonal shading.

The temporal distribution of the different genotypes, variants and clusters in each department is shown in Fig. 3. This figure illustrates consecutive outbreaks with distinct clusters of the GII.4 Den Haag 2006b variant in the nephrology department and both internal medicine departments. It also demonstrates the occurrence of sequences belonging to cluster 1 during a prolonged time period in four different departments. The temporarily related cases in the cardiology department and the internal medicine department 1 (week 5–7/2008) likely represent inter-department spread. During week 13–15/2008, three samples belonging to cluster 1 were taken in three different departments (nephrology, internal medicine 1 and 2), also suggesting inter-department spread.

**Figure 4:**
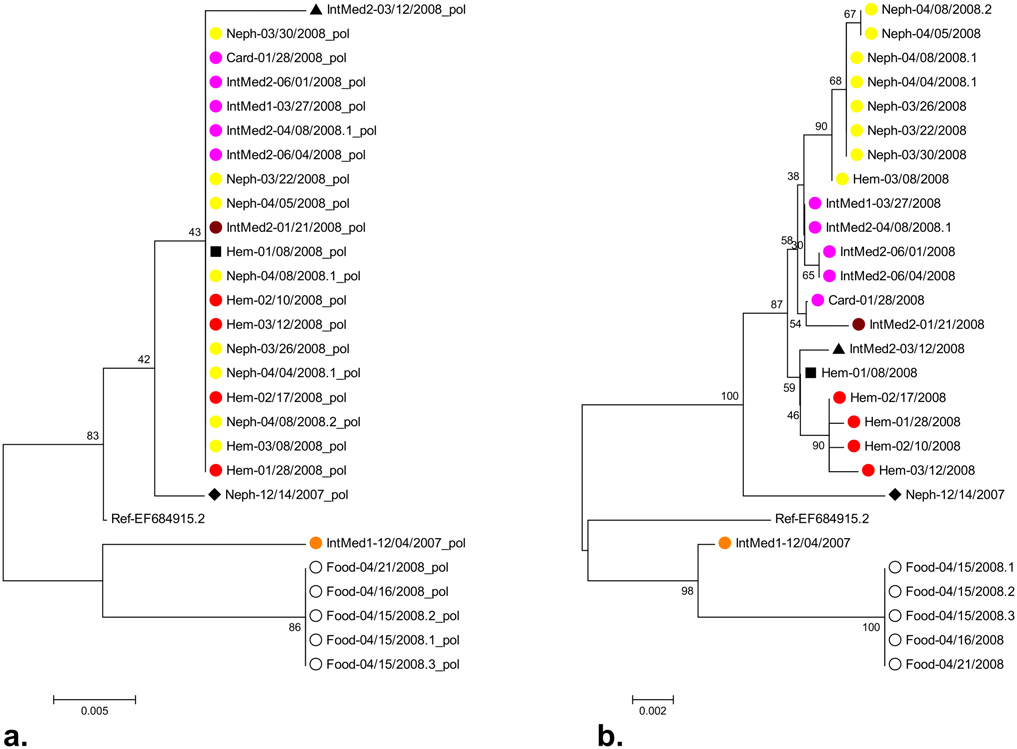
Comparative phylogenetic analysis of polymerase (a.) and capsid (b.) gene sequences. Neighbor Joining trees based on polymerase sequences (a., 226–285 nt) and capsid sequences (b., 1412 nt) of 27 samples from which both sequences were available are shown. Color labels indicate clustering according to the phylogenetic analysis of all capsid sequences shown in Fig. 1 (cluster 1: pink, cluster 2: yellow, cluster 3: brown, cluster 4: red, cluster 5: orange, foodborne cluster 7: empty black circle, singleton 2: black triangle, singleton 3: black square, singleton 4: black diamond.

For 27 samples with a GII.4 variant Den Haag 2006 capsid sequence, polymerase sequences from previous genotyping were also available, with lengths ranging from 226 to 285 nt. Phylogenetic analysis of the polymerase and the capsid sequences of these 27 samples was conducted separately to compare the resolution of these two methods. Nineteen of the 22 samples from inpatients had identical polymerase sequences, whereas phylogenetic analysis of the capsid gene showed that this apparent cluster was comprised of the above described clusters 1, 2, 3 and 4 and singleton 3 (Fig. 4). This demonstrates, as expected, that the longer sequences of the variable capsid gene have a higher discrimination.

### Repeated samples from patients with chronic NoV infection

Paired samples from six patients, all receiving immunosuppressive therapy, were available for sequencing. The samples were taken with an interval of 16 to 77 (median 45) days. All paired sequences were closely related and there was no evidence of reinfection with another NoV strain. However, sequence variation (in 2–13 positions, median 3) was detected between all paired samples and mixed bases were observed in all later samples. In total, mixed bases indicating NoV quasispecies were detected at 37 sites in the paired samples. A significantly higher frequency of mixed bases were observed in the second samples (32 in six 1412 nt sequences) than in the first samples (five mixed bases in six 1412 nt sequences, Chi-squared test, p<0.001).

The nucleotide changes and mixed bases in the sequences from the four patients belonging to cluster 4, together with the remaining sequences of that cluster, are shown in Fig. 2. Sustained nucleotide changes, occurring in more than one patient and suggesting person-to-person transmission after *in vivo* evolution, were found in nine positions.

## Discussion

We used sequencing of a large part of the capsid gene to investigate NoV infections in a university hospital during a nine months’ period in the winter season 2007/2008. Routine polymerase genotyping had shown that the majority of strains belonged to the GII.4 Den Haag 2006b variant and that most of the sequences were identical (Fig. 4). For further outbreak investigation, a method with a higher resolution was needed and substantially longer sequences of the variable capsid gene were therefore examined.

The phylogenetic analysis of the 1412 nt long capsid sequence showed that the accumulation of NoV infections in the hospital was due to several outbreaks mostly confined to one department each. In two clusters (GII.4 Den Haag 2006b clusters 1 and 2), possible inter-department spread was observed. The heterogeneous cluster 1 contained sequences from four different departments over a time span of nearly half a year. One of the later samples in this cluster was taken from a patient without earlier hospital contact within 48 hours after admission, thus indicating a new introduction of the virus from the community into the hospital. However, inter-department spread could have occurred in temporarily related samples from this cluster. An example of this could be the simultaneously occurring norovirus infections in the cardiology department and the internal medicine department 1 in week 5–7/2008. In other temporary and/or locally related cases with sequences differing by no more than one nucleotide the transmission route was unclear. Identical sequences belonging to cluster 1 were for example found in three patients on three different wards in week 13–15/2008. Both nosocomial transmission and repeated introduction from the community could explain these infections. The same is true for the first two samples from cluster 2 which were from patients from two different departments.

In the nephrology department and both internal medicine departments, consecutive outbreaks with distinct clusters of the GII.4 variant Den Haag 2006b were detected, indicating repeated introductions of different strains from the community. As opposed to this, a prolonged outbreak over 12 weeks with the same cluster occurred in the hematology department.

Sequencing of the capsid gene has previously been shown to be helpful for investigating outbreaks. Comparable to our study, which found a presumed larger hospital outbreak to consist of several distinct smaller outbreaks, Xerry et al. [25] could differentiate two distinct simultaneous outbreaks in different departments of the same hospital and Sukhrie et al. [24] found that one of four epidemiological outbreaks involved several unrelated strains. However, in the study of Xerry et al., P2 sequences also detected links between presumably unrelated outbreaks in different departments and even two different hospitals [25]. Similarly, in another study by Sukhrie and coworkers only three out of 14 molecular clusters found by sequencing of the P2 region had been identified through epidemiological investigations, even though the conservative approach of defining a molecular cluster by 100% sequence identity was chosen [28].

The frequency of nucleotide changes found in our study (0 to 12 substitutions per cluster in 1412 nt sequences) is in concordance with previous studies investigating nosocomial NoV GII.4 outbreaks: Dingle et al. showed a diversity of six nucleotide changes in the entire NoV genome in a 1-week outbreak with presumed person-to-person transmission, whereas the strains of an outbreak with a presumed point source only differed by one substitution [26]. Sukhrie et al. found a similar sequence variation of two to 22 nucleotide changes in the P2 domain within prospectively monitored nosocomial outbreaks, where patients and health care workers were sampled once weekly for one to two months [24]. However, in contrast to these and our findings, Xerry et al. described 100% identity in P2 regions of strains in different nosocomial clusters, the largest of which involving four wards in two different hospitals over a seven weeks’ period where 24 samples were examined [25].

A possible epidemiological explanation for outbreaks consisting of identical strains could be a point source such as contaminated food (as seen in the foodborne outbreak in our study) or continuous transmission of the same strain from a contaminated environment, the extent of which is still debated [37]. In our study, no nucleotide changes were found within the sequences in four clusters representing outbreaks confined to one distinct department within a one month’s period (Den Haag 2006b clusters 5 and 6, the Osaka 2007 cluster and the Apeldoorn 2007 cluster). In two clusters (Den Haag 2006b clusters 2 and 3) closely related sequence patterns (one to two nucleotide changes per 1412 nt) were found temporarily related in the same department. Our study does not permit a definite conclusion whether these outbreaks with identical or nearly identical sequences were due to environmental transmission or shorter person-to-person transmissions between individuals with acute infections without evidence of *in vivo* evolution.

In immunocompromised patients, the *in vivo* evolution of the virus leading to the development of quasispecies has been shown [14–16]. In accordance to this, we found the highest sequence diversity in the prolonged outbreak in the hematology department, where the majority of patients was immunosuppressed either due to their underlying disease or induced by chemotherapy. This outbreak (Den Haag 2006b cluster 4) included four of the six patients, where sequences from paired samples were available. The high frequency of mixed bases in these patients’ second samples shows that viral quasispecies had evolved and there was indication for subsequent person-to-person transmission (Fig. 2). The frequencies of sequence variation in paired samples from immunocompromised patients with chronic NoV infection in our study are in accordance with previous studies [16, 26].

Repeated analysis of the data by generation of a Maximum Likelihood tree allocated the sequences to the same clusters (S2 Fig.), demonstrating the method’s robustness despite of the low bootstrap value of cluster 1. However, one of the Den Haag 2006b singleton sequences (“singleton 1”) was obtained from a sample taken on the same day and in the same department as a sample belonging to the heterogeneous cluster 1. As these two sequences only differ by one nucleotide substitution, nosocomial transmission is very likely. This underlines that manual inspection of the sequences, eventually supplemented with epidemiological information, is helpful in interpreting phylogenetic analysis in areas of uncertainty.

This study was designed to investigate sequence variation as an indicator for NoV evolution to study transmission routes. However, further characterization of the nucleotide changes showed interesting findings. Transversions (compared to transitions) and nucleotide changes in the first two codon positions were significantly more common within the P2 region than outside the P2 region, as were non-synonymous substitutions. These findings are in accordance with previous studies and indicate positive selection in this surface-exposed region, which is consistent with its predicted key role for antigenic variation and receptor binding [10, 13, 16, 38]. Five amino acid substitutions were found in key positions of the Den Haag 2006b variant which shows that potentially functional changes in virus—host interacting sites can occur even in a locally and temporarily confined setting.

A drawback of this study is that the available sequences only represent a part of the actual burden of NoV infections. Usually, most samples are taken in the beginning of an outbreak and subsequent cases are diagnosed based on clinical findings. In addition, in our study a substantial part of the samples were not available or sequences could not be obtained. However, as the phylogenetic analysis shows a clear clustering according to department and time of sampling, we feel confident that this bias has not influenced the major findings and conclusions of the study.

This study also lacks information about the genetic variability of strains occurring in the community during the study period. Especially for the interpretation of the heterogeneous Den Haag 2006b cluster 1, this information would have been valuable.

Interestingly, it was difficult to find a clear consensus in the literature about the exact demarcation of the P2 region in NoV GII.4 strains. Several slightly different regions, ranging in length from 125 to 152 amino acids, have been reported [13, 25, 26, 33]. In this study, the definition from a recent article referring to a Den Haag 2006b variant [33] was used. However, the above described characteristics of nucleotide substitutions were still applicable, when using the other cited definitions (data not shown).

In conclusion, sequencing of the capsid gene proved useful for the investigation of a NoV season dominated by the GII.4 variant Den Haag 2006b in one university hospital. Polymerase sequences which were available from genotyping performed continuously during the outbreak were identical. Phylogenetic analysis of the capsid sequences, however, showed that repeated introductions were common, leading to smaller outbreaks, which were most often confined to a single department. However, a few cases of possible inter-department transmission were also detected. If promptly available, these results would have been of value for outbreak management. In this setting, one should have focused on correct isolation measures upon patient admission to prevent subsequent intra-department spread instead of regarding the outbreak as a pan-institutional outbreak. The sustained outbreak on the hematology ward was shown to be due to repeated person-to-person transmission indicating that outbreak management should have focused on isolation measures of chronically infected patients.

## Supporting Information

S1 Table Assignment of accession numbers and sequence names. The accession numbers and sequence names of the 66 capsid sequences and 27 polymerase sequences included in this study are given.
(PDF)

S1 Fig Estimates of evolutionary divergence over sequence pairs between GII.4 variants (a.) and GII.4 Den Haag 2006b clusters (b.). The number of base substitutions per site from averaging over all sequence pairs are shown. Analyses were conducted using the Jukes-Cantor model.
(TIF)

S2 Fig Maximum Likelihood (ML) tree of 53 NoV genotype II.4 variant Den Haag 2006b sequences. A ML tree of all capsid sequences (first sample per patient) was constructed and rooted on the branch between the Den Haag 2006b variant and the Osaka 2007 and Apeldoorn 2007 sequences. The subtree of the 2006b variant is displayed here. Sites with mixed bases were ignored for phylogenetic analysis.
Sequence names represent hospital department (Hem: hematology, Neph: nephrology, IntMed1/2: internal medicine 1/2, Card: cardiology, Food: foodborne outbreak) and date of sampling (mm/dd/yyyy), eventually followed by a serial number to ensure uniqueness.
Sequences from patients with symptom onset within 48 hours after hospital admission are marked with a circle (filled circle: no previous contact to hospital, empty circle: frequent contact to hospital due to hemodialysis). Sequences from the first samples from six patients with persistent diarrhea are marked with a filled triangle. Sequences from samples with an available polymerase gene sequence from routine genotyping (n = 27) are marked with asterisks.
(TIF)
