# Supplementary material for: Sequence Analysis of the Capsid Gene during a Genotype II.4 Dominated Norovirus Season in One University Hospital: Identification of Possible Transmission Routes
Source: PLoS One. 2015 Jan 15;10(1):e0115331. doi: 10.1371/journal.pone.0115331 (PMC4295850; doi:10.1371/journal.pone.0115331)
Supplement: S1 Table — The accession numbers and sequence names of the 66 capsid sequences and 27 polymerase sequences included in this study are given. (PDF) [file pone.0115331.s001.pdf]

Capsid sequence		Polymerase sequence		
**Sequence name**	**GenBank Accession No**	**Sequence name in article**	**GenBank sequence name**	**GenBank Accession No**
Neph-12/14/2007	KJ144938	Neph-12/14/2007_pol	Neph12-14-2007_pol	KJ956701
IntMed1-12/27/2007	KJ144939			
IntMed1-12/04/2007	KJ144940	IntMed1-12/04/2007_pol	IntMed1-12-04-2007_pol	KJ956726
IntMed1-11/20/2007	KJ144941			
IntMed1-11/28/2007	KJ144942			
Neph-12/18/2007	KJ144943			
Neph-04/04/2008.1	KJ144944	Neph-04/04/2008.1_pol	Neph04-04-2008.1_pol	KJ956702
Hem-04/03/2008	KJ144945			
IntMed1-01/16/2008	KJ144946			
Card-02/08/2008	KJ144947			
Hem-02/08/2008	KJ144948			
Neph-02/13/2008	KJ144949			
Card-02/14/2008	KJ144950			
Card-02/15/2008	KJ144951			
Neph-03/26/2008	KJ144952	Neph-03/26/2008_pol	Neph03-26-2008_pol	KJ956704
Food-04/16/2008	KJ144953	Food-04/16/2008_pol	Food04-16-2008_pol	KJ956719
Food-04/15/2008.1	KJ144954	Food-04/15/2008.1_pol	Food04-15-2008.1_pol	KJ956720
Neph-04/24/2008	KJ144955			
Hem-04/30/2008	KJ144956			
Hem-05/07/2008	KJ144957			
IntMed1-01/31/2008	KJ144958			
Hem-03/05/2008	KJ144959			
Hem-03/06/2008	KJ144960			
Other-01/10/2008	KJ144961			
Hem-01/26/2008	KJ144962			
IntMed2-01/21/2008	KJ144963	IntMed2-01/21/2008_pol	IntMed2-01-21-2008_pol	KJ956724
Hem-01/28/2008	KJ144964	Hem-01/28/2008_pol	Hem01-28-2008_pol	KJ956708
Hem-02/10/2008	KJ144965	Hem-02/10/2008_pol	Hem02-10-2008_pol	KJ956723
IntMed2-02/04/2008	KJ144966			
Hem-02/15/2008	KJ144967			
Hem-02/17/2008	KJ144968	Hem-02/17/2008_pol	Hem02-17-2008_pol	KJ956703
Neph-02/22/2008	KJ144969			
Hem-02/22/2008.1	KJ144970			
Hem-02/22/2008.2	KJ144971			
Neph-03/29/2008	KJ144972			
Neph-03/30/2008	KJ144973	Neph-03/30/2008_pol	Neph03-30-2008_pol	KJ956707
Food-04/21/2008	KJ144974	Food-04/21/2008_pol	Food04-21-2008_pol	KJ956722
IntMed2-06/01/2008	KJ144975	IntMed2-06/01/2008_pol	IntMed2-06-01-2008_pol	KJ956705
Neph-04/04/2008.2	KJ144976			
Hem-01/08/2008	KJ144977	Hem-01/08/2008_pol	Hem01-08-2008_pol	KJ956725
IntMed1-01/14/2008.1	KJ144978			
IntMed1-01/14/2008.2	KJ144979			
Hem-01/27/2008	KJ144980			
Card-01/28/2008	KJ144981	Card-01/28/2008_pol	Card01-28-2008_pol	KJ956709
Hem-02/09/2008	KJ144982			
Hem-02/18/2008	KJ144983			
Hem-03/23/2008	KJ144984			
Neph-03/22/2008	KJ144985	Neph-03/22/2008_pol	Neph03-22-2008_pol	KJ956714
Neph-04/05/2008	KJ144986	Neph-04/05/2008_pol	Neph04-05-2008_pol	KJ956713
IntMed2-04/08/2008.1	KJ144987	IntMed2-04/08/2008.1_pol	IntMed2-04-08-2008.1_pol	KJ956727
Hem-04/14/2008	KJ144988			
Food-04/15/2008.2	KJ144989	Food-04/15/2008.2_pol	Food04-15-2008.2_pol	KJ956718
Food-04/15/2008.3	KJ144990	Food-04/15/2008.3_pol	Food04-15-2008.3_pol	KJ956721
Hem-05/02/2008	KJ144991			
IntMed2-05/09/2008	KJ144992			
Hem-03/08/2008	KJ144993	Hem-03/08/2008_pol	Hem03-08-2008_pol	KJ956711
IntMed2-04/08/2008.2	KJ144994			
IntMed2-06/04/2008	KJ144995	IntMed2-06/04/2008_pol	IntMed2-06-04-2008_pol	KJ956710
Neph-04/08/2008.1	KJ144996	Neph-04/08/2008.1_pol	Neph04-08-2008.1_pol	KJ956716
Neph-04/08/2008.2	KJ144997	Neph-04/08/2008.2_pol	Neph04-08-2008.2_pol	KJ956706
Hem-04/19/2008	KJ144998			
IntMed1-01/29/2008	KJ144999			
IntMed2-02/12/2008	KJ145000			
Hem-03/12/2008	KJ145001	Hem-03/12/2008_pol	Hem03-12-2008_pol	KJ956712
IntMed2-03/12/2008	KJ145002	IntMed2-03/12/2008_pol	IntMed2-03-12-2008_pol	KJ956717
IntMed1-03/27/2008	KJ145003	IntMed1-03/27/2008_pol	IntMed1-03-27-2008_pol	KJ956715

Table S1. Assignment of accession numbers and sequence names.
The accession numbers and sequence names of the 66 capsid sequences and 27 polymerase sequences included in this study are given.
