# Supplementary figures and images for: Sequence Analysis of the Capsid Gene during a Genotype II.4 Dominated Norovirus Season in One University Hospital: Identification of Possible Transmission Routes
Source: PLoS One. 2015 Jan 15;10(1):e0115331. doi: 10.1371/journal.pone.0115331 (PMC4295850; doi:10.1371/journal.pone.0115331)

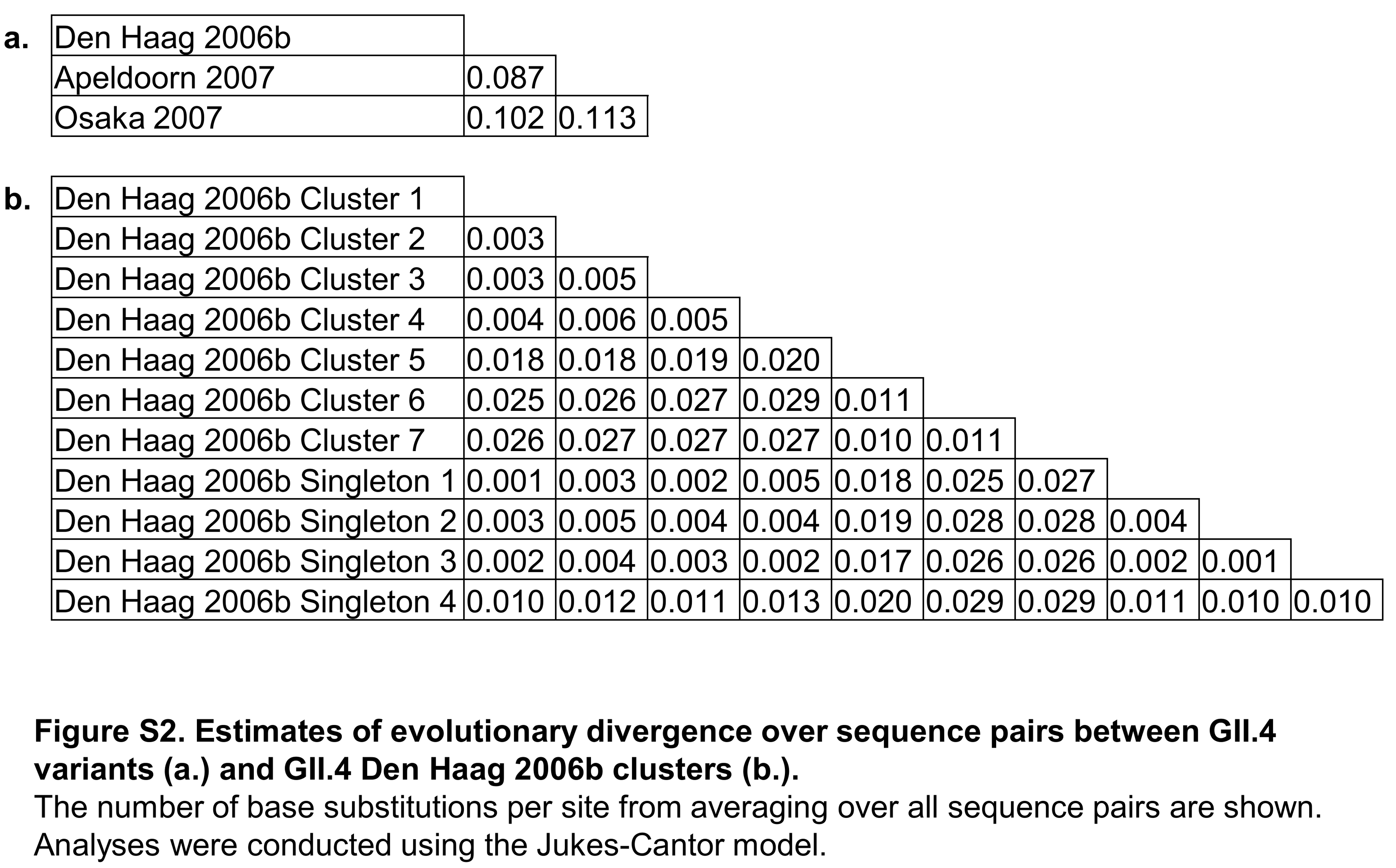

Supplement: S1 Fig — The number of base substitutions per site from averaging over all sequence pairs are shown. Analyses were conducted using the Jukes-Cantor model. (TIF) [file pone.0115331.s002.tif]

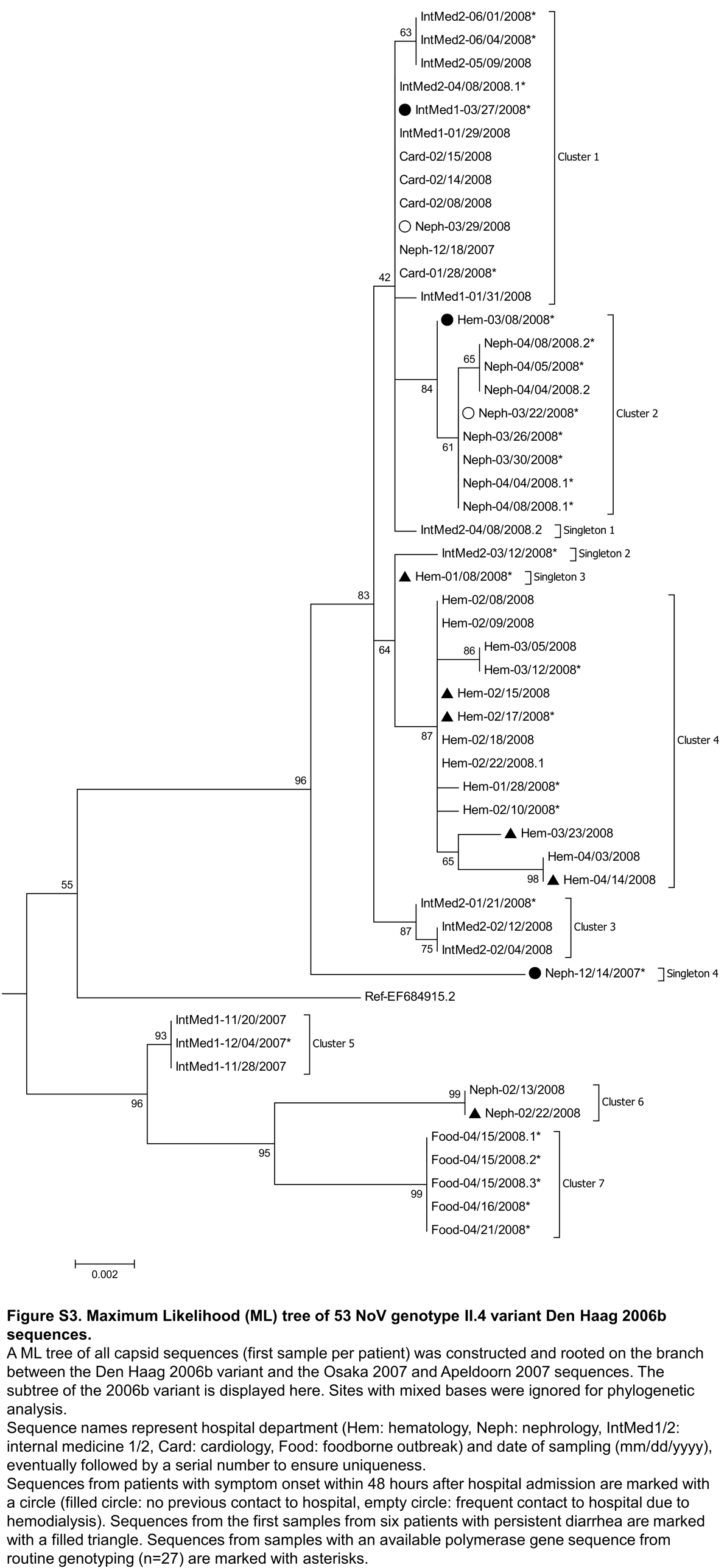

Supplement: S2 Fig — A ML tree of all capsid sequences (first sample per patient) was constructed and rooted on the branch between the Den Haag 2006b variant and the Osaka 2007 and Apeldoorn 2007 sequences. The subtree of the 2006b variant is displayed here. Sites with mixed bases were ignored for phylogenetic analysis. Sequence names represent hospital department (Hem: hematology, Neph: nephrology, IntMed1/2: internal medicine 1/2, Card: cardiology, Food: foodborne outbreak) and date of sampling (mm/dd/yyyy), eventually followed by a serial number to ensure uniqueness. Sequences from patients with symptom onset within 48 hours after hospital admission are marked with a circle (filled circle: no previous contact to hospital, empty circle: frequent contact to hospital due to hemodialysis). Sequences from the first samples from six patients with persistent diarrhea are marked with a filled triangle. Sequences from samples with an available polymerase gene sequence from routine genotyping (n = 27) are marked with asterisks. (TIF) [file pone.0115331.s003.tif]
